# Supplementary material for: Descent trajectory reconstruction and landing site positioning of Chang’E-4 on the lunar farside
Source: Nat Commun. 2019 Sep 24;10:4229. doi: 10.1038/s41467-019-12278-3 (PMC6760200; doi:10.1038/s41467-019-12278-3)
Supplement: Supplementary file 1 — Supplementary Information [file 41467_2019_12278_MOESM1_ESM.pdf]

# SUPPLEMENTARY INFORMATION

# Descent trajectory reconstruction and landing site positioning of Chang'E-4 on the lunar farside

**Jianjun Liu et al**

## CONTENTS

**Supplementary Figures (1-9)**

**Supplementary Tables (1-5)**

## Supplementary Figures

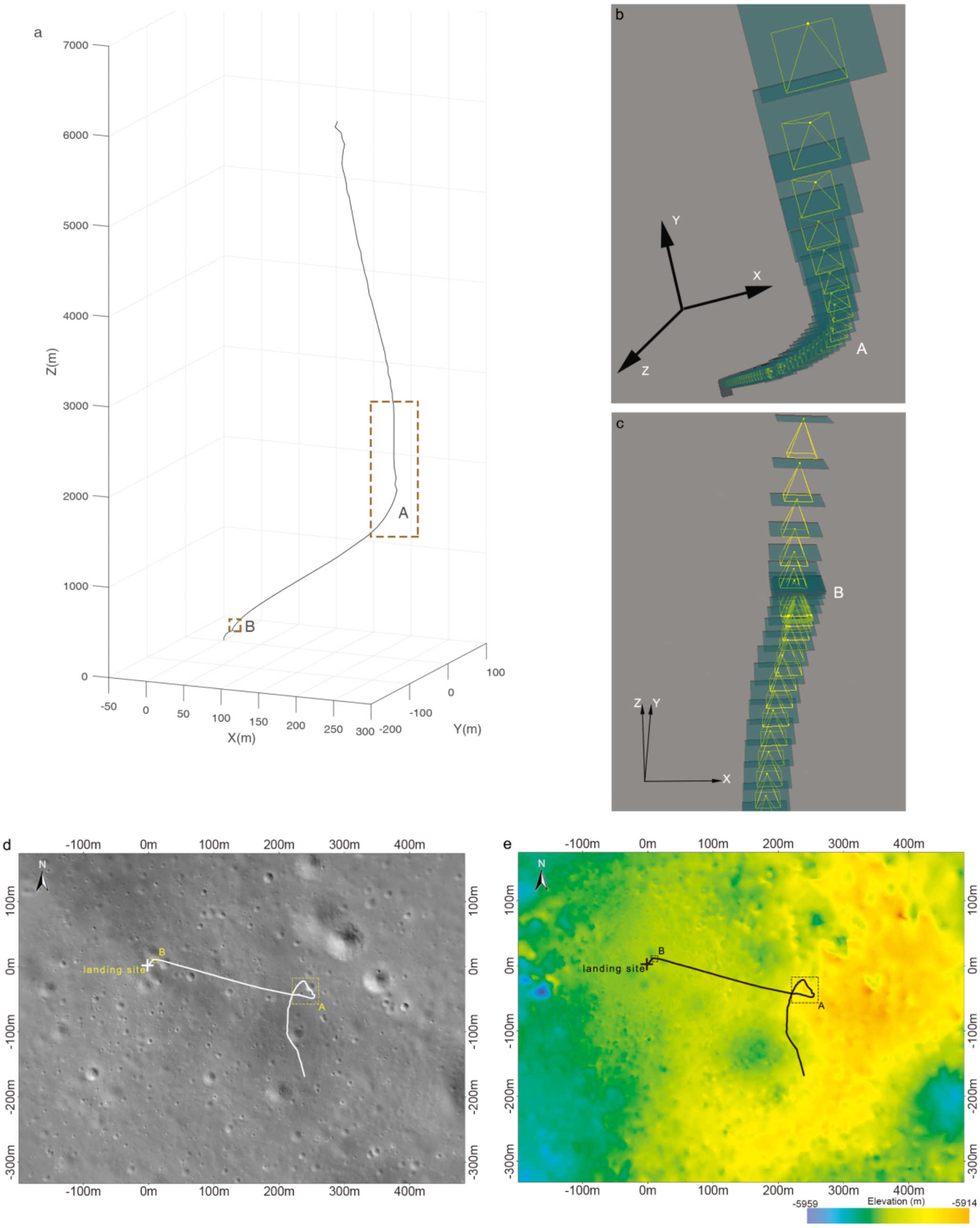


**Supplementary Figure 1. CE-4 powered descent trajectory.** The + is the identified landing location. (**a**) CE-4 descent trajectory from the altitude of 6000 m to the lunar surface; (**b**) and (**c**) are zoomed images of the descent trajectories of A and B, respectively. The green box represents the position of the LCAM focal plane, and the yellow cone represents the field of view of the LCAM. (**d**) The solid white line is the projection of the descent trajectory on the LCAM DOM. (**e**) The solid black line is the projection of the descent trajectory on the LCAM DEM. The coordinate system in this figure is the tangent plane coordinate system of the landing site. This system uses the location of the landing site as the coordinate origin, with its X and Y axes pointing towards the geographical east and the north of the landing site, respectively. The Z axis, X axis, and Y axis form a right-hand system, with the Z axis pointing to the zenith direction of the landing site.

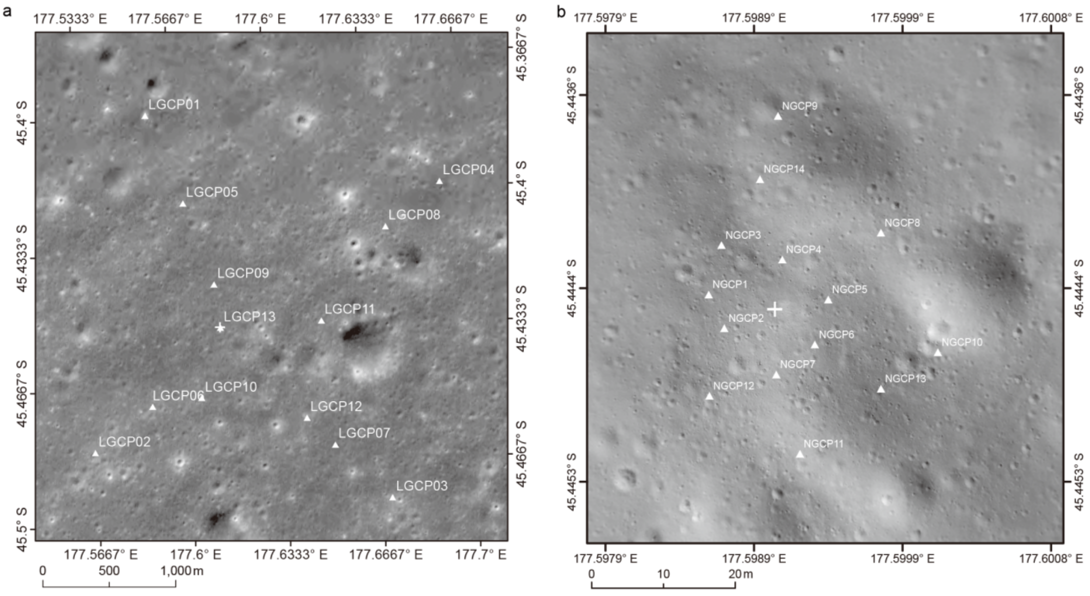


**Supplementary Figure 2. GCPs map for photogrammetric adjustment processing.** (a) GCPs on the CE2TMap2015 DOM for the LCAM image adjustment, marked as LGCPs, in which LGCP13 is the vertical GCP; (b) GCPs on the LCAM DOM for the NCAM image adjustment, marked as NGCPs. The + is the landing site.

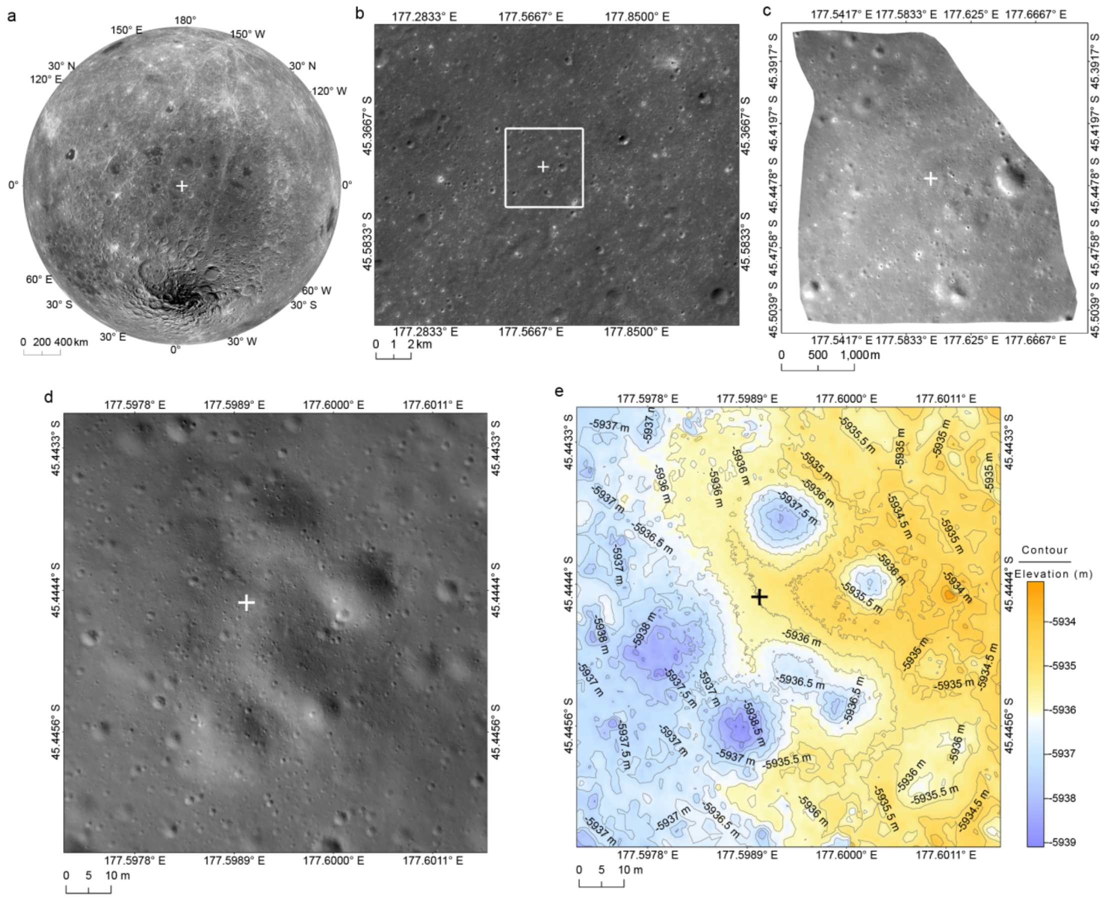


**Supplementary Figure 3. Location of the CE-4 landing site.** The + is the identified landing location. (**a**) and (**b**) are the CE-2 DOMs, using the CE-4 landing site as the projection center of the azimuth projection. (**c**) is the LCAM DOM with a resolution of 5 m/p. (**d**) is the LCAM DOM, which is generated by the LCAM images with resolutions from 21.4 cm/p to 0.2 cm/p and is uniformly resampled at 5 cm/p during mapping. (**e**) is a shaded-relief map with contour line produced by the LCAM DEM, which area and resolution are the same as in (**d**).

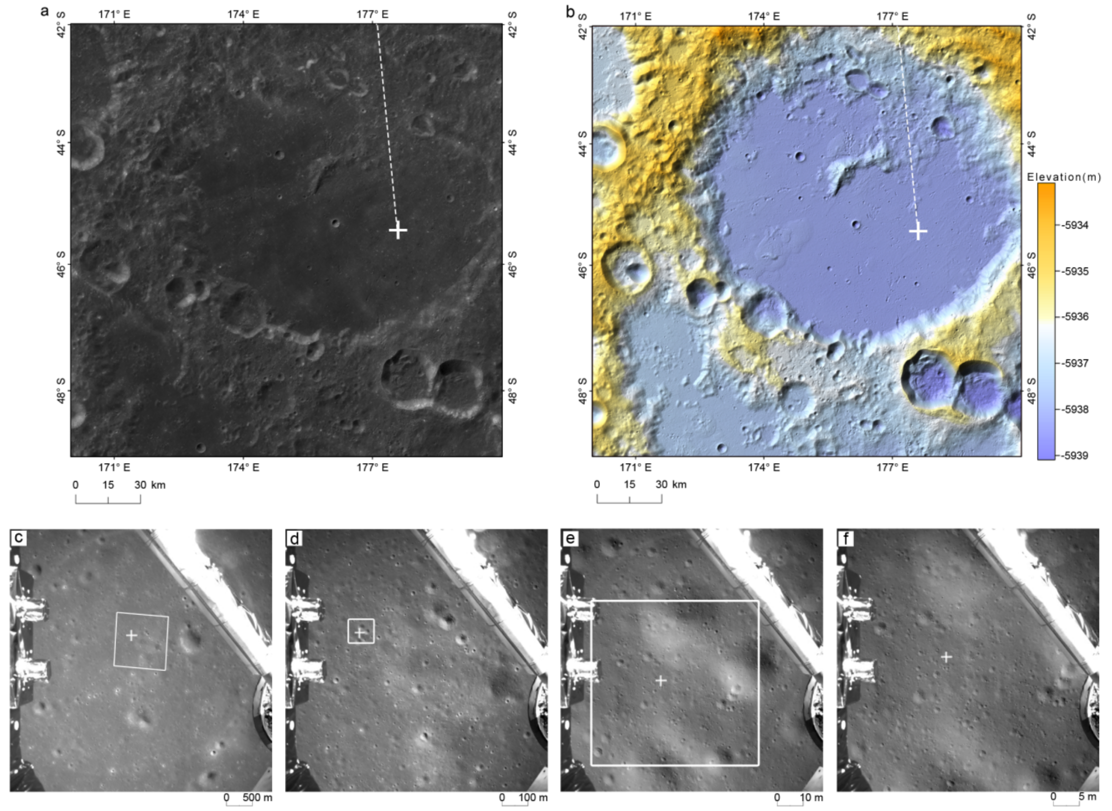


**Supplementary Figure 4. The CE-4 landing site on CE2TMap2015 and CE-4 LCAM images.** The + is the identified landing location. (**a**) Landing site on the Chang'E-2 DOM. (**b**) Landing site on a shaded-relief map made by the Chang'E-2 DEM. The projection is the positive-axis isometric Mercator projection, which has a standard parallel at 45°S. White dotted line represents the approach trajectory of CE-4; (**c**), (**d**), (**e**) and (**f**) are the LCAM images with spatial resolutions of 5 m/p, 1 m/p, 10 cm/p and 5 cm/p, respectively. The white rectangles in (**c**), (**d**), and **(e)** represent the borders of (**d**), (**e**), and **(f)**, respectively.

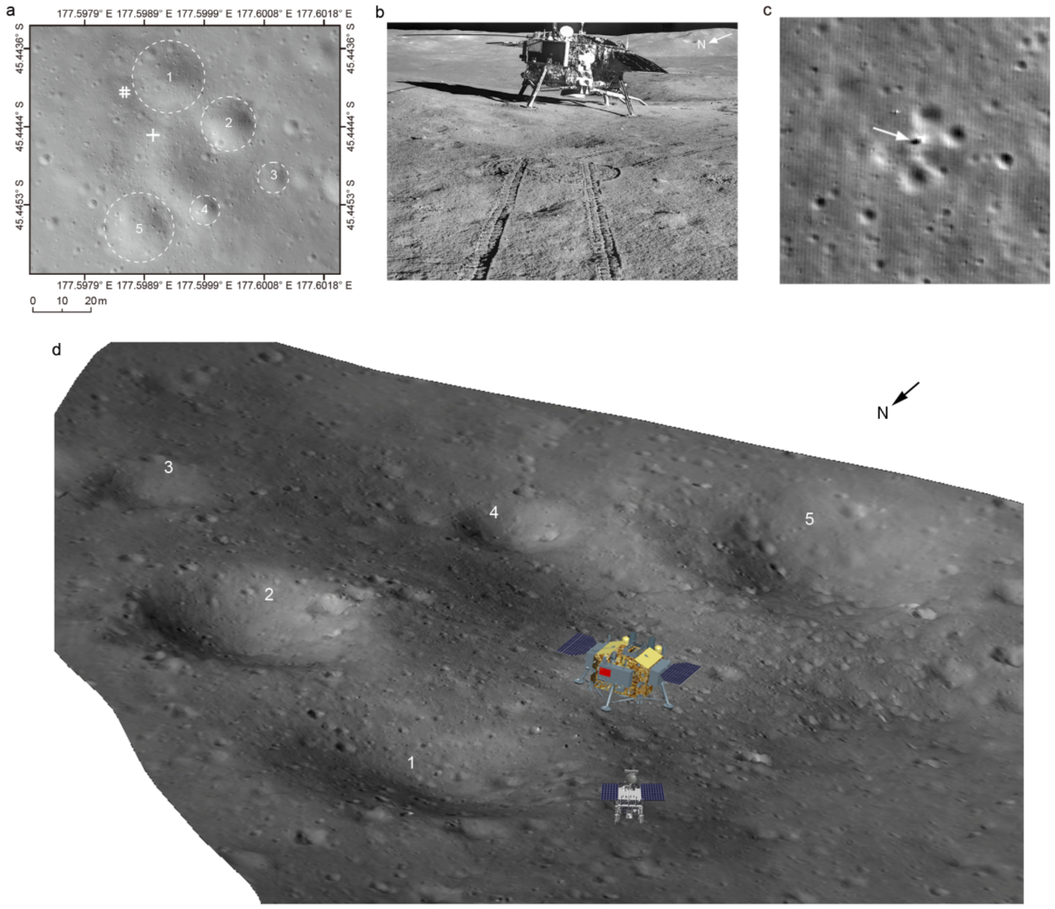


**Supplementary Figure 5. Position of the CE-4 landing site.** (**a**) The CE-4 landing site on the 5 cm/p resolution LCAM DOM, in which the + is the identified landing location and the # is the location of Yutu-2 on 12 January 2019. Five prominent craters around the landing site are marked. (**b**) CE-4 lander image obtained from the northwest of the landing site by the Yutu-2 PCAM at the location labeled in (**a**). (**c**) LRO NAC image of the CE-4 landing site collected on 1 February 2019 (M1303640934LR), in which the arrow indicates the location of CE-4 landing site. (**d**) Three-dimensional landscape map of the landing site generated by the DEM and DOM data through reconstruction of CE-4 LCAM sequential images acquired within 100 m above the lunar surface, in which the location of CE-4 lander and Yutu-2 is the same as labeled in (a). While landing position was determined from LCAM orbital data (**a**), the orientation of the lander was determined using PCAM image taken from the ground (**b**). Prominent craters are also marked in (**d**), as in (**a**).

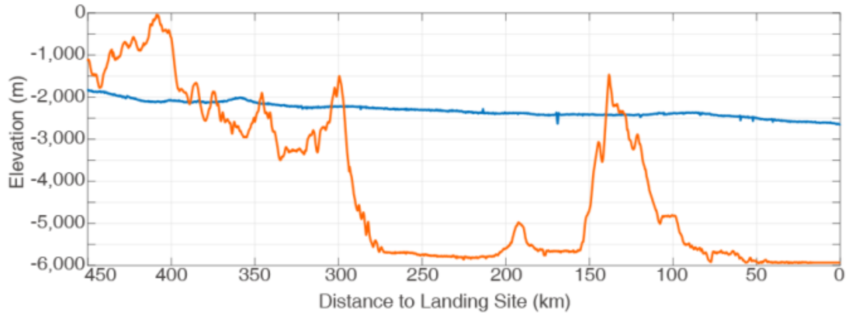


**Supplementary Figure 6.** Elevation variations of the lunar terrain along the CE-3 and CE-4 nadir flight traces. The blue and orange lines represent the elevation variation for the CE-3 and CE-4 nadir flight trace, respectively. Elevation is given with respect to the Moon's spheroid with a radius of 1737.4 km.

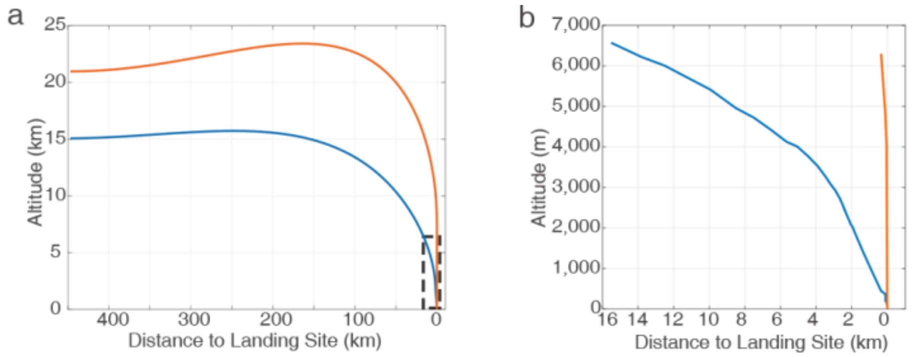


**Supplementary Figure 7. Comparison of powered descent trajectory between the CE-3 and CE-4.** The blue and orange lines represent trajectories of the CE-3 and CE-4, respectively. (**a)** The designed trajectories of the entire powered descent, in which the marked rectangle corresponds to designed trajectories below the altitude of ~6km; (**b)** Magnified portion of the reconstructed trajectory below the altitude of ~6km. Altitude is given with respect to final landing site level.

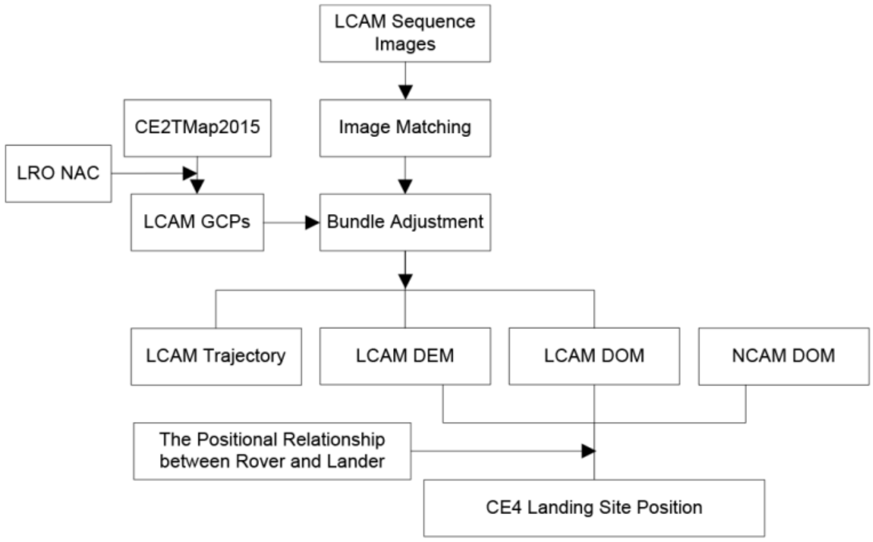


**Supplementary Figure 8. Data processing flowchart.**

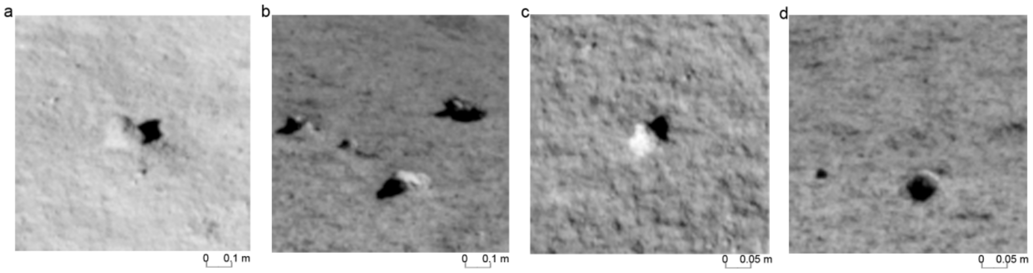


**Supplementary Figure 9. Small rocks used as GCPs.** (**a**)-(**d**) are stones in the NCAM image that can also be easily identified in the LCAM DOM.

## Supplementary Tables

**Supplementary Table 1. Information on the CE-4 Landing Site**

| Element | Description |
|---|---|
| Landing site geographic coordinates | 177.5991°E，45.4446°S，-5935 m. (The position uncertainty relative to CE2TMap2015 is meter level; See the Methods for details) |
| Coordinate system | The lunar coordinate is based on the mean Earth/polar axis coordinate system. The reference surface of the elevation is the surface of the Moon's spheroid with a radius of 1737.4 km, and the reference origin is the mass center of the Moon. |
| Landing site location in the images* | (1) In the CE2Tmap2015 DOM with a resolution of 7 m, landing site pixel coordinate (line 13602, column 21647)<br>(2) In the LCAM image with a resolution of 5 m, landing site pixel coordinate (line 412, column 472)<br>(3) In the LCAM image with a resolution of 1 m, landing site pixel coordinate (line 403, column 288)<br>(4) In the LCAM image with a resolution of 10 cm, landing site pixel coordinate (line 592, column 389)<br>(5) In the LCAM image with a resolution of 5 cm, landing site pixel coordinate (line 500, column 426) |

*The subdivision number for the CE2TMap2015 DOM and DEM with a resolution of 7 m is K136 in this study (Fig. 4 (a) and Fig. 4 (b)). The corresponding file names for LCAM 5 m, 1 m, 10 cm and 5 cm resolution images are as follows:

CE4_LCAM_20190103022303_1740_08.tif (Fig. 5 (c))，

CE4_LCAM_20190103022414_2444_08.tif (Fig. 5 (d))，

CE4_LCAM_20190103022500_2906_08.tif (Fig. 5 (e))

and CE4_LCAM_20190103022525_3160_08.tif (Fig. 5 (f) ).

**Supplementary Table 2. The Performance Parameters of the LCAM**

| No. | Name | Performance Parameters |
|---|---|---|
| 1. | Wavelength range (nm) | 419～777 |
| 2. | Field of view (°) | 45.3°×45.3° |
| 3. | Focal Length (mm) | 8.5 |
| 4. | Effective pixel numbers | 1024×1024 |
| 5. | Pixel size on Focal plane (μm) | 6.7 |
| 6. | Frame rate (fps) | 10 |
| 7. | Quantized value (bit) | 8 |
| 8. | Data compression ratio | 8:1 |

**Supplementary Table 3. The Performance Parameters of the NCAM**

| No. | Name | Performance Parameters |
|---|---|---|
| 1. | Focal Length (mm) | Left camera: 17.69<br>Right camera: 17.98 |
| 2. | Effective pixel numbers | 1024×1024 |
| 3. | Pixel size on Focal plane (μm) | 15 |
| 4. | Baseline length (mm) | 269.69 |
| 5. | Quantized value (bit) | 8 |

**Supplementary Table 4. Horizontal and Vertical Deviations of the GCPs for the LCAM**

| Name | RMS of Reprojection Error (pixel) | Horizontal Error (m) | Vertical Error (m) |
|---|---|---|---|
| **LGCP01** | 0.35 | 1.522 | -2.319 |
| **LGCP02** | 0.74 | 2.560 | 0.504 |
| **LGCP03** | 0.21 | 1.327 | -1.528 |
| **LGCP04** | 0.72 | 2.933 | 1.365 |
| **LGCP05** | 0.55 | 0.897 | 0.807 |
| **LGCP06** | 0.67 | 1.565 | -0.642 |
| **LGCP07** | 0.72 | 1.685 | -0.513 |
| **LGCP08** | 0.23 | 0.710 | 0.610 |
| **LGCP09** | 0.93 | 0.795 | 0.799 |
| **LGCP10** | 1.08 | 1.048 | -0.788 |
| **LGCP11** | 0.75 | 0.855 | 0.420 |
| **LGCP12** | 0.84 | 0.865 | -0.483 |
| **LGCP13** | 0.11 | / | 0.065 |
| **Global RMS** | 0.61 | 0.715 | 1.040 |

**Supplementary Table 5. Horizontal and Vertical Deviations of the GCPs for the NCAM**

| Name | RMS of the Reprojection Error (pixel) | Horizontal Error (m) | Vertical Error (m) |
|---|---|---|---|
| **NGCP01** | 0.077 | 0.053 | -0.038 |
| **NGCP02** | 0.006 | 0.034 | 0.038 |
| **NGCP03** | 0.007 | 0.014 | -0.037 |
| **NGCP04** | 0.010 | 0.032 | 0.048 |
| **NGCP05** | 0.005 | 0.021 | 0.017 |
| **NGCP06** | 0.007 | 0.028 | -0.065 |
| **NGCP07** | 0.003 | 0.027 | -0.004 |
| **NGCP08** | 0.007 | 0.040 | -0.009 |
| **NGCP09** | 0.013 | 0.075 | 0.013 |
| **NGCP10** | 0.005 | 0.049 | -0.027 |
| **NGCP11** | 0.005 | 0.031 | 0.014 |
| **NGCP12** | 0.005 | 0.020 | 0.001 |
| **NGCP13** | 0.005 | 0.015 | 0.040 |
| **NGCP14** | 0.017 | 0.014 | 0.015 |
| **Global RMS** | 0.012 | 0.017 | 0.031 |
